# Supplementary material for: Layer-specific cortical dynamics during transitions from error monitoring to decision execution in reversal learning
Source: Commun Biol. 2025 Dec 11;9:69. doi: 10.1038/s42003-025-09336-6 (PMC12808145; doi:10.1038/s42003-025-09336-6)
Supplement: Supplementary file 2 — Supplemental Information [file 42003_2025_9336_MOESM2_ESM.pdf]

Supplemental Information for Acun, E. et al. (COMMSBIO-25-2889)

Layer-Specific Cortical Dynamics During Transitions from Error Monitoring to Decision Execution in Reversal Learning

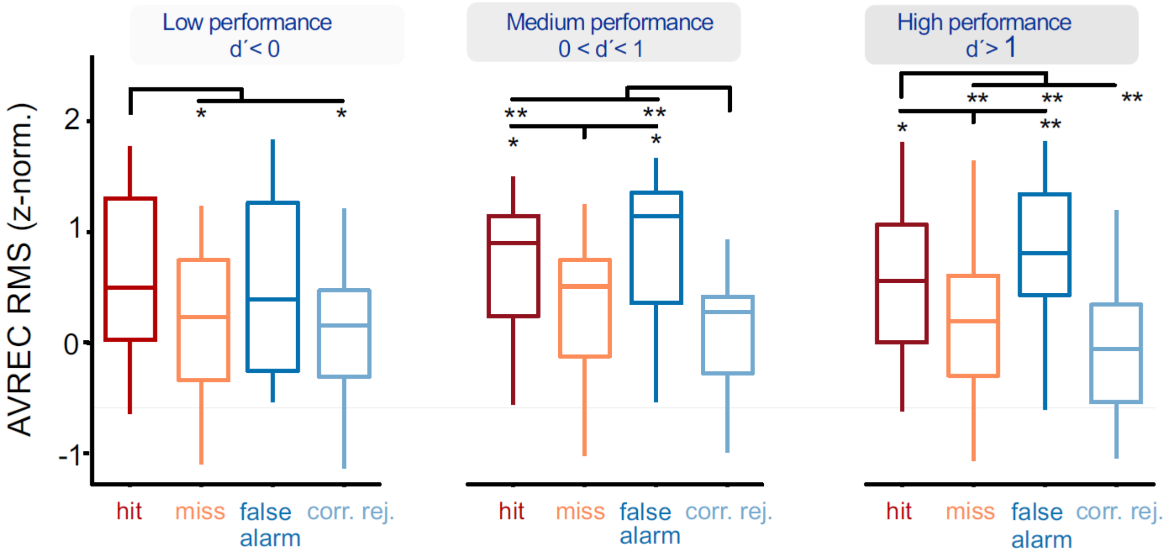

Figure S1: ANOVA analysis of the AVREC waveform before decision making across different layers and performance levels. AVREC RMS values (500 ms window after stimulus onset preceding the reaction time) are plotted with respect to behavioral choices (hit, miss, false alarm, correct rejection) across all reversal blocks (reversals 1-4) and split by performance levels based on the sensitivity index (d'). Low performance levels (d' < 0), intermediate performance levels (0 < d' < 1), and high performance levels (d' > 1), are plotted from left to right. Box-plots show median, interquartile range, full data range, and outliers, with significant differences indicated by a two-way rmANOVA with Holm-corrected post-hoc tests. Asterisks indicate statistical significance of differences between groups (*p ≤ 0.05 or **p ≤ 0.01).
